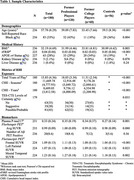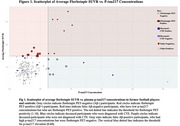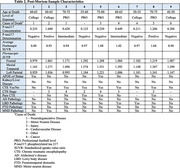# The utility of plasma *p*‐tau217 in former American football players at risk for chronic traumatic encephalopathy

**DOI:** 10.1002/alz70856_105546

**Published:** 2026-01-09

**Authors:** Annalise E. Miner, Nicholas J. Ashton, Henrik Zetterberg, Kaj Blennow, Jenna R. Groh, Yorghos Tripodis, Charles Adler, Laura Balcer, Charles B. Bernick, Elaine R. Peskind, Breton M. Asken, Jeremy A. Tanner, Gil D. Rabinovici, Sarah J Banks, William B. Barr, Jennifer V. Wethe, Robert C. Cantu, David W. Dodick, Jesse Mez, Joseph N. Palmisano, Brett Martin, Thor D. Stein, Ann C. McKee, Jeffrey L. Cummings, Martha E. Shenton, Eric M. Reiman, Robert A. Stern, Michael L Alosco

**Affiliations:** ^1^ Boston University Alzheimer's Disease Research Center, Boston, MA, USA; ^2^ Banner Alzheimer's Institute, Phoenix, AZ, USA; ^3^ Wallenberg Centre for Molecular and Translational Medicine, University of Gothenburg, Gothenburg, Sweden; ^4^ Hong Kong Center for Neurodegenerative Diseases, Hong Kong, Science Park, China; ^5^ Department of Psychiatry and Neurochemistry, Institute of Neuroscience and Physiology, the Sahlgrenska Academy, University of Gothenburg, Molndal, Sweden; ^6^ Clinical Neurochemistry Laboratory, Sahlgrenska University Hospital, Mölndal, Västra Götalands län, Sweden; ^7^ Department of Neurodegenerative Disease, UCL Institute of Neurology, Queen Square, London, United Kingdom; ^8^ Wisconsin Alzheimer's Disease Research Center, University of Wisconsin School of Medicine and Public Health, Madison, WI, USA; ^9^ UK Dementia Research Institute, University College London, London, United Kingdom; ^10^ Paris Brain Institute, ICM, Pitié‐Salpêtrière Hospital, Sorbonne University, Paris, France; ^11^ Neurodegenerative Disorder Research Center, Institute on Aging and Brain Disorders, University of Science and Technology of China and First Affiliated Hospital of USTC, Heifei, China; ^12^ Clinical Neurochemistry Laboratory, Sahlgrenska University Hospital, Mölndal, Sweden; ^13^ Department of Psychiatry and Neurochemistry, University of Gothenburg, Mölndal, Sweden; ^14^ Boston University School of Public Health, Boston, MA, USA; ^15^ Parkinson's Disease and Movement Disorders Center, Mayo Clinic, Scottsdale, AZ, USA; ^16^ NYU Grossman School of Medicine, New York, NY, USA; ^17^ Lou Ruvo Center for Brain Health, Cleveland Clinic, Las Vegas, NV, USA; ^18^ Veterans Affairs Puget Sound Health Care System, Seattle, WA, USA; ^19^ University of Washington School of Medicine, Seattle, WA, USA; ^20^ Department of Clinical and Health Psychology (B.M.A.), University of Florida, Gainesville, FL, USA; ^21^ 1Florida Alzheimer's Disease Research Center, Gainesville, FL, USA; ^22^ University of Texas Health San Antonio, San Antonio, TX, USA; ^23^ Department of Radiology and Biomedical Imaging, University of California San Francisco, San Francisco, CA, USA; ^24^ UCSF Alzheimer's Disease Research Center, San Francisco, CA, USA; ^25^ Memory and Aging Center, Weill Institute for Neurosciences, University of California San Francisco, San Francisco, CA, USA; ^26^ University of California, San Diego, La Jolla, CA, USA; ^27^ NYU Langone Health, New York City, NY, USA; ^28^ Mayo Clinic, Scottsdale, AZ, USA; ^29^ Department of Neurology, Boston University Chobanian & Avedisian School of Medicin, Boston, MA, USA; ^30^ Boston University Chronic Traumatic Encephalopathy Center, Boston, MA, USA; ^31^ Department of Veterans Affairs Medical Center, Bedford, MA, USA; ^32^ Boston University Chronic Traumatic Encephalopathy Center, Boston University Chobanian & Avedisian School of Medicine, Boston, MA, USA; ^33^ Boston University Chobanian & Avedisian School of Medicine, Boston, MA, USA; ^34^ Department of Pathology and Laboratory Medicine, Boston University Chobanian & Avedisian School of Medicine, Boston, MA, USA; ^35^ Department of Neurology, Boston University Chobanian & Avedisian School of Medicine, Boston, MA, USA; ^36^ Chambers‐Grundy Center for Transformative Neuroscience, Department of Brain Health, School of Integrated Health Sciences, University of Nevada Las Vegas, Las Vegas, NV, USA; ^37^ Brigham and Women's Hospital, Boston, MA, USA; ^38^ University of Arizona, Phoenix, AZ, USA; ^39^ Arizona State University, Phoenix, AZ, USA

## Abstract

**Background:**

*In vivo* biomarkers that can detect long‐term neuropathologies from repetitive head impact (RHI) exposure are needed, especially for the neurodegenerative tauopathy chronic traumatic encephalopathy (CTE). Here, we evaluated plasma *p*‐tau217 as a potential biomarker for CTE *p*‐tau pathology, and examined the concordance between plasma *p*‐tau217 and Aβ pathology in an at‐risk for CTE sample.

**Method:**

The sample included 180 male former football players (120 professional, 60 college), and 56 asymptomatic men without RHI (i.e., controls). Participants completed blood draws, 18F‐florbetapir (Aβ+=SUVR≥1.10), and 18F‐flortaucipir PET. Traumatic encephalopathy syndrome (TES) diagnoses were made. Single molecule array for plasma *p*‐tau217 (ALZpath) was performed (≥0.6 cutoff used to maximize sensitivity). Nine participants had post‐mortem tissue. ANCOVA examined group differences in *p*‐tau217 (football vs controls; TES‐CTE no, TES‐CTE suggestive, TES‐CTE possible/probable). Multivariable regression models tested associations between *p*‐tau217 and florbetapir/flortaucipir PET. Covariates included age, race and *APOE e4*.

**Result:**

Sample characteristics are in Table 1. *p*‐tau217 concentrations were higher in former football players compared to controls (est. marginal mean difference=‐0.217, *p* = 0.005). There were no group differences in Aβ‐PET SUVR. No differences were found across TES‐CTE certainty levels. In football players, higher *p*‐tau217 was associated with higher Aβ‐PET SUVR (B=1.380, 95%CI[0.597‐2.155], *p* = 0.001) but not when Aβ+ (*n* = 17) participants and those with kidney/liver disease (*n* = 5) were excluded. Aβ+ participants had the highest *p*‐tau217 (Figure 1). When compared against Aβ‐PET, several false Aβ‐positives (high *p*‐tau217, Aβ‐) were identified, including one extreme outlier (assay related) and a cluster of Aβ‐ participants with *p*‐tau217 between 0.60–1.0. There were no associations with flortaucipir SUVR (frontal, mesial temporal, left parietal). Two extreme *p*‐tau217 outliers had autopsy‐confirmed CTE stage III (AD‐, Table 2). Of the remaining donors, all were AD‐ and four had CTE (stages II‐IV) with ptau217 between 0.125‐0.449.

**Conclusion:**

Plasma *p*‐tau217 has usefulness in quantifying Aβ pathology but restricted utility for detection of CTE. In this at‐risk for CTE sample, *p*‐tau217 and Aβ‐PET were associated at the group level. At the individual level, false Aβ‐positives (and negatives) existed, including Aβ‐ participants with high *p*‐tau217. We will explore whether this discrepancy is due to disease or peripheral interference with the N‐terminal binding in *p*‐tau assays.